# Can melatonin reduce the severity of post-COVID-19 syndrome?

**DOI:** 10.17179/excli2023-5864

**Published:** 2023-02-02

**Authors:** Amine Souissi, Ismail Dergaa, Mohamed Romdhani, Amine Ghram, Khadijeh Irandoust, Karim Chamari, Helmi Ben Saad

**Affiliations:** 1 Université de Sousse, Faculté de Médecine de Sousse, Hôpital Farhat HACHED, Laboratoire de Recherche (Insuffisance Cardiaque, LR12SP09), Sousse, Tunisie; 2 Primary Health Care Corporation (PHCC), Doha, P.O. Box 26555, Qatar; 3 Research Unit: Physical Activity, Sport, and Health, UR18JS01, National Observatory of Sport, Tunis, Tunisia; 4 Motricité-Interactions-Performance, MIP, UR4334, Le Mans Université, Le Mans, France; 5 Department of Sport Sciences, Imam Khomeini International University, Qazvin, Iran; 6 Aspetar, Orthopedic and Sports Medicine Hospital, FIFA Medical Center of Excellence, Doha, Qatar

**Keywords:** antioxidant status, cytokines, dose, free radicals, stress

## Abstract

This short review aimed at ***(i)*** providing an update on the health benefits associated with melatonin supplementation, while ***(ii)*** considering future potential research directions concerning melatonin supplementation use relative to Coronavirus disease of 2019 (COVID-19). A narrative review of the literature was undertaken to ascertain the effect of exogenous melatonin administration on humans. Night-time melatonin administration has a positive impact on human physiology and mental health. Indeed, melatonin ***(i)*** modulates the circadian components of the sleep-wake cycle; ***(ii)*** improves sleep efficiency and mood status; ***(iii)*** improves insulin sensitivity; and ***(iv)*** reduces inflammatory markers and oxidative stress. Melatonin has also remarkable neuroprotective and cardioprotective effects and may therefore prevent deterioration caused by COVID-19. We suggest that melatonin could be used as a potential therapy in the post-COVID-19 syndrome, and therefore call for action the research community to investigate on the potential use of exogenous melatonin to enhance the quality of life in patients with post-COVID-19 syndrome.

See also Figure 1(Fig. 1).

## Introduction

Melatonin is an indoleamine produced by the pineal gland in humans (Ackermann and Stehle, 2006[4]; Arendt, 2006[16]; Zawilska et al., 2006[160]). Its regulation is impacted by dark-light and both seasonal, and lunar cycles (Arendt and Broadway, 1987[17]; Dergaa et al., 2019[40]; Dergaa et al., 2021[42]; Nelson et al., 2015[109]). Melatonin secretion decreases progressively with advancing age, resulting in a reduction in sleep duration (Iguchi et al., 1982[69]). Endogenous melatonin levels are known to decrease substantially after 40 years, and this decline can be causally linked to lowered sleep efficacy (Haimov et al., 1994[66]; Pierpaoli et al., 1995[117]), eventually affecting physical and mental health (Karasek, 2004[73]). Exogenous melatonin supplementation may potentially have beneficial effects on sleep as well as age-related diseases (Karasek, 2004[73]). Therefore, the potential role of melatonin supplementation in the prevention and/or treatment of insomnia and age-related diseases is promising. Importantly, at least for short regimens, melatonin supplementation in humans is safe, as demonstrated by preclinical and clinical data (Colella et al., 2016[30]; Minich et al., 2022[103]; Sánchez-Barceló et al., 2010[131]). 

Interestingly, the high levels of melatonin concentration in children may be a contributing factor to their high protection against the coronavirus disease of 2019 (COVID-19) (Shneider et al., 2020[134]). Indeed, endogenous melatonin seems to play a key role in suppressing COVID-19 infections. Therefore, melatonin supplementation may have a variety of health benefits, including improved quality of life and cost savings in healthcare, and may help prevent complications associated with COVID-19 (Giri et al., 2021[62]; Ramos et al., 2021[120]). However, for years, data obtained in animals have been uncritically extrapolated to humans (Kennaway, 2019[74]). 

Thus, the purposes of this narrative review were to ***(i)*** summarize recent findings regarding melatonin supplementation in humans and its clinical implications; and ***(ii)*** provide some suggestions on what future research in the field should focus on.

## Treatment of Sleep Disorders with Exogenous Melatonin

The primary physiological function of melatonin is to inform the human body about the diurnal light/dark cycle and to synchronize the central and peripheral oscillators located in tissues and organs (Khullar, 2012[75]; Rong et al., 2020[129]). Melatonin secretion is widely regarded as the best known indicator of the circadian body clock's state (Folkard, 2008[56]). Importantly, the circadian clock system is critical for homeostasis and human health maintenance (Pickel and Sung, 2020[116]; Van Someren, 2000[152]). Specifically, melatonin exerts chronobiotic effects by stimulating G protein-dependent receptors types 1 and 2 (Khullar, 2012[75]; Slominski et al., 2012[135]). This function is critical because disruptions in circadian rhythm can increase the risk of metabolic, cardiovascular, and/or mental diseases, as well as result in poorer health outcomes (Aly and Rizk, 2018[9]; Hacışevki and Baba, 2018[65]). 

Consequently, exogenous melatonin supplementation has been proven to be effective at restoring sleep-wake cycles and enhancing sleep quality, and is frequently used as a medication to treat sleep disorders, such as sleeplessness and jet lag syndrome (Laudon and Frydman-Marom, 2014[82]). Melatonin increases sleepiness and shortens sleep onset latency by exerting its sedative effect on the central nervous system (Gandolfi et al., 2020[58]; Marseglia et al., 2015[98]; Souissi et al., 2020[140], 2022[138]).

## Can Melatonin Improve Cognitive Function?

It has been shown that 1 mg of exogenous melatonin administered at night may be effective in improving sleep quality, memory, and cognitive functioning in elderly people (Peck et al., 2004[114]). It seems that exogenous melatonin is also a useful therapeutic agent in the treatment of mental impairment associated with aging (Peck et al., 2004[114]). In fact, exogenous melatonin is particularly effective in reversing age-related cognitive decline when decreased endogenous melatonin secretion has been detected in patients (Pandi-Perumal et al., 2005[111]).

Endogenous melatonin secretion is decreased in people of advanced age, for instance (Burgess and Fogg, 2008[24]), or lower in those who are blind (Warman et al., 2011[156]), or in patients with neurodegenerative disorders (Rajpoot, 2020[119]). Consequently, melatonin treatment in these populations resulted in a significant improvement of the clinical and neurophysiological aspects of rapid eye movement and sleep behavior disorders in blind populations (Warman et al., 2011[156]), and in elderly patients with underlying neurodegenerative disorders (St Louis and Boeve, 2017[142]; Videnovic et al., 2020[154]). In the same context, meta-analysis studies reported that melatonin treatment improved sleep quality in patients with Alzheimer and Parkinson diseases, and it may be used as an exclusive or adjunctive therapy in patients with neurodegenerative disorders and associated behavioral and cognitive function disorders (Ferracioli-Oda et al., 2013[54]; Zhang et al., 2016[163]).

## What Is the Beneficial Role of Melatonin in Physical Performance?

The association between physical exercise and melatonin secretion has been well discussed (Escames et al., 2012[49]). Melatonin concentration increases temporarily in the blood during exercise (Carr et al., 1981[27]; Ronkainen et al., 1986[130]; Theron et al., 1984[148]), suggesting that melatonin plays a role during physical exercise (Souissi et al., 2022[137]). However, melatonin supplementation may be beneficial or useless depending on the type of physical exercise (López-Flores et al., 2018[89]). 

Sleep and recovery are key components of athletes' health and performance enhancements (Gander et al., 2008[57]; Walsh et al., 2021[155]). According to a recent study (Kruk et al., 2021[78]), athletes frequently use sleep-enhancing supplements. In this regard, melatonin is among the most commonly used supplement due to its wide-range effects on the organism, including, but not limited to, its antioxidant properties that protect muscles and mitochondria from oxidative stress (Liesa and Shirihai, 2013[86]). Furthermore, exogenous melatonin has been proven to be an effective antioxidant and anti-inflammatory agent, eventually maintaining mitochondrial function (Hu et al., 2019[68]), as well as muscular strength and adaptability during heavy exercise (Borges et al., 2015[22]; Ochoa et al., 2011[110]; Trionfante et al., 2017[151]). It has been recently shown that melatonin supplementation during congested training periods can enhance antioxidant status and glucose resistance in different types of training, including soccer training camps and resistance training athletes (Farjallah et al., 2022[52]; Leonardo-Mendonça et al., 2017[84]; Souissi et al., 2022[136]).

Endurance exercise consumes glucose, resulting in a decrease in muscle and liver glycogen stores (Trefts et al., 2015[150]). For ATP synthesis, melatonin can partly shift glucose metabolism from anaerobic glycolysis to aerobic mitochondrial oxidative phosphorylation, and consequently result in decreased lactate production (Mazepa et al., 1999[100]; Sayed et al., 2018[133]). Therefore, pre-exercise melatonin administration can enhance lipid utilization as a substrate energy source (Mazepa et al., 1999[100]; Souissi et al., 2022[136]; Trionfante et al., 2017[151]). Future studies should investigate the relationship between melatonin secretion/supplementation and the body mass index, and why not considering melatonin as a potential way to help patients with obesity to optimize the outcome of their exercise programs.

## Can Melatonin Improve Mood Status?

Environmental disruptions of circadian rhythms, including the sleep-wake cycle, can result in mood-related perturbations in susceptible people (Altun and Ugur‐Altun, 2007[7]; Germain and Kupfer, 2008[59]; Ghazel et al., 2022[60]; Monteleone and Maj, 2008[105]), and in athletes (Romdhani et al., 2019[127]). Indeed, numerous preclinical and clinical findings have suggested a strong link between circadian rhythms, melatonin secretion dysregulation, and mood regulation (Altun and Ugur‐Altun, 2007[7]; Etain et al., 2011[50]; Germain and Kupfer, 2008[59]; Lanfumey et al., 2013[81]; Lerner and Nordlund, 1978[85]; McClung, 2011[101]; Monteleone and Maj, 2008[105]; Monteleone et al., 2011[106]; Munk-Jørgensen, 2014[108]). 

Exogenous melatonin ingestion has a favorable clinical response for reducing sleep difficulties or improving health problems associated with circadian rhythms disruption, which may be prevalent in the symptomatology of mood disorders (Coogan and Thome, 2011[31]; Maldonado et al., 2009[94]). A prospective observational study identified that exogenous melatonin consumption improved sleep and mood (Livianos et al., 2012[88]). This suggests that its use may be part of a therapeutic toolkit for the treatment of anxiety and depression related to sleep deprivation (Quera Salva and Hartley, 2012[118]; Robillard et al., 2018[124]). In this context, a recent study revealed that salivary melatonin levels were negatively correlated with the severity of depression (Sundberg et al., 2016[146]) and suggested that melatonin could become a clinically useful biomarker of stress, anxiety and depression (Chojnowska et al., 2021[29]; Kudo et al., 2021[79]; Sundberg et al., 2016[146]). The effects of melatonin ingestion on depressive symptoms may represent an interesting area of research in the future.

## Can Melatonin Reduce Cardiovascular Risk?

Low blood melatonin levels have been found in a variety of clinical diseases, such as arterial hypertension (Dominguez-Rodriguez et al., 2014[46]; Jonas et al., 2003[71]; Koziróg et al., 2011[77]), heart failure (Dominguez-Rodriguez et al., 2016[45]; Dzida et al., 2013[48]; Girotti et al., 2003[63]; Kimak et al., 2014[76]), ischemic and heart diseases (Altun et al., 2002[8]; Brugger et al., 1995[23]; Domínguez‐Rodríguez et al., 2002[44]), and cardiovascular related risk conditions such as diabetes mellitus and obesity (Mäntele et al., 2012[97]; McMullan et al., 2013[102]). In this context, exogenous melatonin has been shown to be beneficial to reduce the internal carotid arteries' pulsatility index and blood clotting (Del Zar et al., 1990[33][34]), and to decrease catecholamine levels in the blood (Arangino et al., 1999[15]; Souissi et al., 2021[139]). Indeed, a negative correlation between endogenous melatonin levels and cardiovascular diseases has been reported (Dominguez‐Rodriguez et al., 2010[47]). The potential therapeutic involvement of melatonin in the pathophysiology of coronary artery disease is being increasingly acknowledged (Jiki et al., 2018[70]; Pandi-Perumal et al., 2016[112]). Furthermore, due to its direct free radical scavenging activity, melatonin appears to have cardioprotective benefits (Dominguez-Rodriguez, 2012[43]; Paulis and Šimko, 2007[113]; Reiter et al., 2010[123]; Sun et al., 2016[145]). The cardiovascular protective action of melatonin supplementation is particularly promising for cardiovascular disorders (Jiki et al., 2018[70]) and exercise-induced cardiovascular fatigue (Souissi et al., 2021[139]) amongst others.

Additionally, we would like to highlight that the sudden cardiac arrests caused by COVID-19 infection depend primarily on the damage inflammation and the cytokine storm induced by the host immune reaction (Tan and Reiter, 2022[147]; Zhang et al., 2020[161]). In this case, melatonin could be used to downregulate the overreaction of the immune system, potentially suppressing/dampening the inflammation and reducing the risk of mortality (Tan and Reiter, 2022[147]). Larger clinical trials are therefore needed to determine the efficacy of melatonin supplementation as a novel preventive intervention in cardiovascular disorders (in the pathophysiology of coronary artery disorder, arterial hypertension, congestive heart failure, COVID-19, and cardiovascular fatigue induced by exercise) in humans.

## Can Melatonin Be Utilized to Treat Respiratory Disease and Viral Infections?

Melatonin has been successfully used to treat respiratory disease and viral infections (Reiter et al., 2020[122]), with its beneficial effects on acute respiratory stress caused by viruses and bacteria being identified (Wu et al., 2019[158]; Yip et al., 2013[159]). Although melatonin is not viricidal, it has indirect anti-viral actions (Reiter et al., 2020[122]) owing to its antioxidative, anti-inflammatory, and immune-enhancing properties (Anderson et al., 2015[13]; Boga et al., 2012[20]; Lee and Glickman, 2021[83]; Reiter et al., 2020[121]). Melatonin use resulted in lowered viremia, decreased viral load, and decreased paralysis and death (Bonilla et al., 2004[21]). Moreover, melatonin has been proven to suppress the effects of viral infections in several conditions (Bahrampour Juybari et al., 2020[19]). In previous respiratory syncytial virus models, melatonin ingestion resulted in a downregulation of acute lung oxidative injury, pro-inflammatory cytokine release, and inflammatory cell recruitment (Zhang et al., 2020[162]). These findings, along with those summarized by Reiter et al. (2020[122]), support the rationale use of melatonin supplementation for respiratory viral diseases. 

## Can Melatonin Boost the Immune Response?

The ability of melatonin to influence the immune response is one of its most intriguing properties (Carrillo-Vico et al., 2013[28]; Kurhaluk and Tkachenko, 2020[80]). Some decades ago, the first evidence that melatonin could boost antibody production and reverse the immunosuppressive impact of corticosteroids and/or acute stress was reported (Maestroni et al., 1987[91]; Maestroni, 2001[92]). The immunoregulatory role of melatonin has recently been well established (Kurhaluk and Tkachenko, 2020[80]; Mańka and Majewska, 2016[96]). Melatonin may operate as an immunological buffer, boosting the immune response in basal or immunosuppressive situations or acting as an anti-inflammatory agent in the context of excessive immune responses (Carrillo-Vico et al., 2013[28]; Mortezaee et al., 2019[107]). Although, it has been recently shown that melatonin could be used to treat some autoimmune diseases, such as multiple sclerosis (Farez et al., 2016[51]) and autoimmune thyroid diseases (D'Angelo et al., 2016[32]), more investigations are warranted to confirm its potential promising therapeutic use (Zhao et al., 2019[164]).

Interestingly, exogenous melatonin treatment may significantly enhance the strength and persistence of the immunological response elicited by the severe COVID-19 (Wichniak et al., 2021[157]). Additionally, it has been shown that the antioxidant properties of melatonin and its pleiotropic effect on the immune system may help reduce some of the deleterious side effects of the COVID-19 vaccination (Maestroni, 2020[90]). It would be possible that melatonin treatment could be more beneficial in elderly people with sleep disorders. So far, the available information suggests that melatonin has a broad capacity to increase both cell-mediated and humoral immune responses regardless of sex or age (Maestroni, 2020[90]).

## Can Melatonin Aid Mitigate the Harmful Effects of the COVID-19 Epidemic?

COVID-19, designated a public health emergency and a global threat of international concern by the World Health Organization, is one of the most worrisome diseases in recent history, with a clear 2020-lockdowns disruption observed in most countries globally (Dergaa et al., 2022[41][39]; Ghram et al., 2021[61]; Mohammed, 2020[104]; Varma et al., 2021[153]). As of January 23, 2022, severe acute respiratory syndrome coronavirus 2 is estimated to have infected globally approximately 663640386 people so far, with 6713093 estimated deaths (https://covid19.who.int/). The stringent public health measures, although effective in reducing person-to-person transmission of COVID-19 (Dergaa et al., 2021[36], 2022[37][38][41][39]) have been shown to negatively impact individuals' lifestyle behaviors (*eg*; physical activity levels, sleep/wake behaviors, diet) (Ammar et al., 2020[10], 2021[11]; Dergaa et al., 2022[37][41]; Romdhani et al., 2022[125][126][128]; Trabelsi et al., 2021[149]), their mental wellbeing and mood state (Akbari et al., 2021[6]). 

Since, endogenous melatonin levels were reduced in patients with COVID-19 infection (Anderson and Reiter, 2020[14]; Attademo and Bernardini, 2021[18]), melatonin has been researched for its potential role in COVID-19 infected patients (Golombek et al., 2022[64]; Zhang et al., 2020[162]). Indeed, melatonin treatment may help mitigate the harmful effects of the COVID-19 epidemic on sleep and mental health (Lee and Glickman, 2021[83]; Wichniak et al., 2021[157]). In addition, the anti-inflammation, antioxidant, and immune enhancing actions of melatonin potential support its potential attenuation of COVID-19 infection (Zhang et al., 2020[162]). Melatonin seems to play a key role in suppressing COVID-19 infection (Martín Giménez et al., 2020[99]). The results of the research projects regarding the melatonin effect on COVID-19 patients are indeed very promising.

## Can Melatonin Reduce the Severity of Post-COVID-19 Syndrome?

Importantly, it has been shown that more than 60 % of COVID-19 survivors experienced post-COVID-19 syndrome (PC19S) (Fernández-de-Las-Peñas et al., 2021[53]). However, contrary to the symptoms and complications of acute COVID-19 that are well established, the sequelae induced by the PC19S need more studies (Malik et al., 2022[95]). So far, patients with PC19Ss have been reported to have persistent symptoms and a poor quality of life (Malik et al., 2022[95]). The exact pathophysiological mechanisms explaining the PC19S are not well understood due to their multifactorial properties. However, since the major serious persistent symptoms highlighted after the infections were sleep disturbance, fatigue, anxiety, depression and/or mental impairment (Malik et al., 2022[95]), one could speculate that endogenous melatonin disturbance is a potential factor contributing to the long-term consequences of COVID-19 infection (Magdy et al., 2022[93]).

Furthermore, it has been recently shown that severe PC19S was associated with radiological evidence of cardiac damage (myocarditis: cause of mortality) (Dennis et al., 2021[35]). Mechanisms explaining myocarditis in patients with PC19S remain unclear (Ackermann et al., 2020[5]; Dennis et al., 2021[35]). However, it is evident that myocarditis has been associated with several factors, particularly with older age (Dennis et al., 2021[35]). In fact, aging is associated with increased oxidative stress (Abete et al., 1999[3]; Ferrucci and Fabbri, 2018[55]), and decreased endogenous protective mechanisms (Abete et al., 1997[1], 2001[2]), including decreased melatonin production (Dominguez‐Rodriguez et al., 2010[47]). Briefly, inflammaging (chronic inflammation in aging) is the main factor of myocarditis (Ferrucci and Fabbri, 2018[55]; Soysal et al., 2020[141]). Therefore, inflammaging associated with PC19S are potential factors for increasing the risk of myocarditis and weakening the immune system efficiency (Soysal et al., 2020[141]).

Melatonin ingestion could reduce the severity of PC19S-inducing myocarditis by attenuating aging and inhibiting the production of inflammatory cytokines (Su et al., 2022[144]). Furthermore, it has been shown that melatonin supplementation significantly improved coxsackie-virus B3-inducing myocarditis in mouse hearts (Sang et al., 2018[132]). Sang et al. (2018[132]) suggested that melatonin could be a promising new therapeutic approach against viral myocarditis. It would be interesting in the future to assay the effect of melatonin supplementation against COVID-19-inducing myocarditis in human hearts.

On the other hand, numerous studies have demonstrated the beneficial effects of exogenous melatonin on mental health and sleep (Lim et al., 2022[87]). Effective sleep disturbance management and early sensorium correction are reported to be critical in preventing post-intensive care syndrome and lowering morbidity (Hashmi et al., 2017[67]). Although evidence regarding the impact of exogenous melatonin on PC19S is lacking, its efficacy and safety have been repeatedly demonstrated in humans (Buscemi et al., 2006[25]), and we expect that its use by patients with PC19Ss would be highly beneficial. The present review is therefore a call to action for public health to explore the effect of exogenous melatonin on PC19S patients. 

Finally, we highlight that exogenous melatonin may present some side effects for some populations (pregnant and breast-feeding women) (Andersen et al., 2016[12]), and should not be consumed at high doses and/or for long time as we do not know the long-term endocrine effects of melatonin administration (Minich et al., 2022[103]). Therefore, it is important that we suggest some recommendations based on expert knowledge and scientific research for optimizing health and offer concrete steps for increasing endogenous melatonin (Table 1(Tab. 1); References in Table 1: Cajochen et al., 1997[26]; Carr et al., 1981[27]; Kałużna-Czaplińska et al., 2019;[72] Peuhkuri et al., 2012[115]; Strassman et al., 1989[143]; Theron et al., 1984[148]).

## Conclusions

Exogenous melatonin supplementation may improve sleep efficiency, mood status, and cognitive performance. In addition, it may have potential therapeutic use in mental health and cardiovascular disorders. At present, melatonin is known to potentially attenuate the COVID-19 infection through its anti-inflammatory, antioxidant, and immune-enhancing properties. We call for research on the potential beneficial use of melatonin in patients with PC19S.

## Notes

Mohamed Romdhani and Amine Ghram contributed equally as third author.

Karim Chamari and Helmi Ben Saad contributed equally as last author.

## Conflict of interest

The authors declare that they have no competing interests. 

## Figures and Tables

**Table 1:**
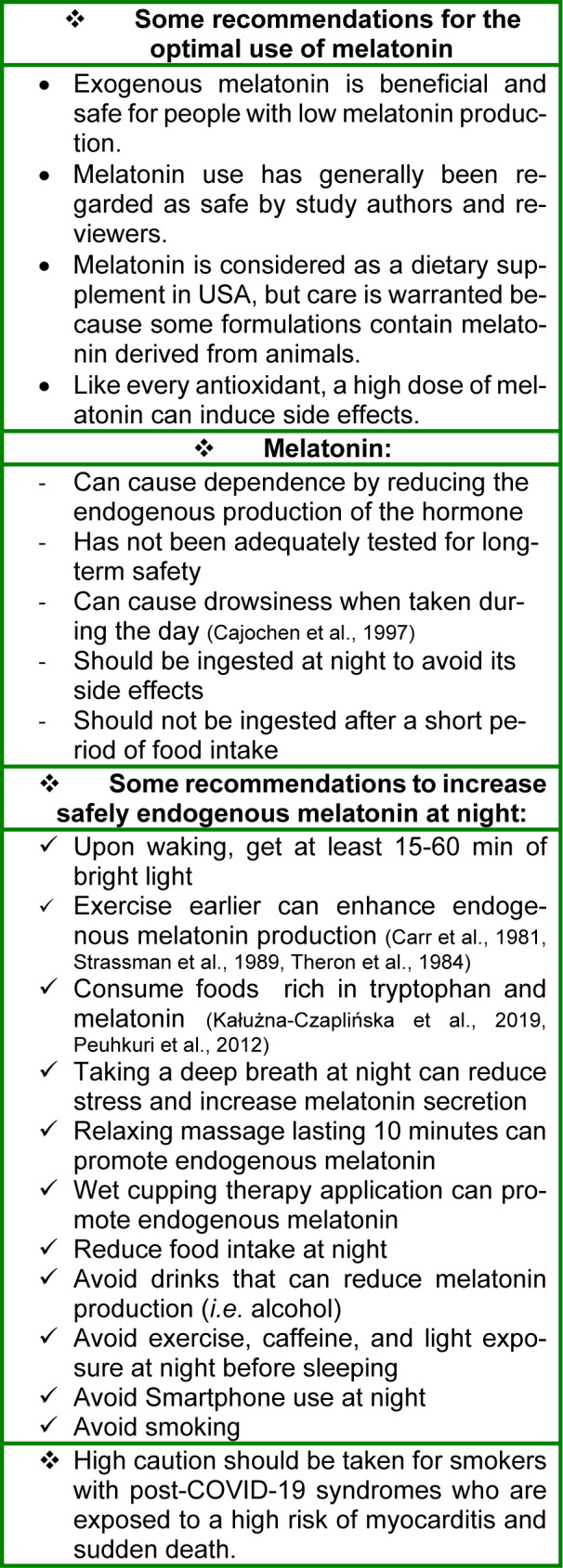
Fight Coronavirus disease of 2019 (COVID-19) and boost immunity by increasing endogenous melatonin levels. Concrete tips for increasing endogenous melatonin and promoting sleep.

**Figure 1:**
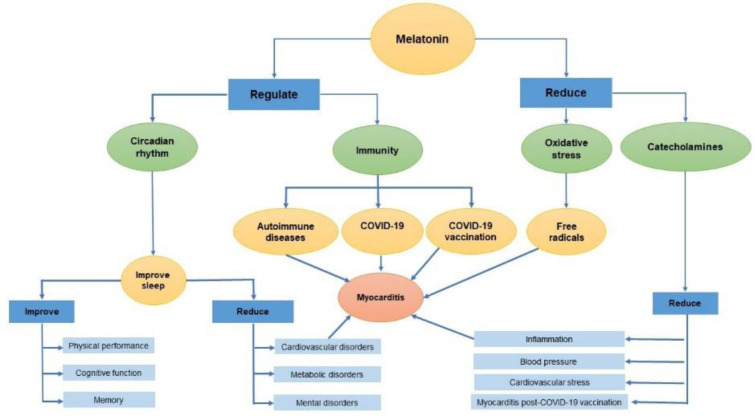
Graphical abstract
